# The CRBN, CUL4A and DDB1 Expression Predicts the Response to Immunomodulatory Drugs and Survival of Multiple Myeloma Patients

**DOI:** 10.3390/jcm10122683

**Published:** 2021-06-18

**Authors:** Joanna Barankiewicz, Anna Szumera-Ciećkiewicz, Aleksander Salomon-Perzyński, Paulina Wieszczy, Agata Malenda, Filip Garbicz, Monika Prochorec-Sobieszek, Irena Misiewicz-Krzemińska, Przemysław Juszczyński, Ewa Lech-Marańda

**Affiliations:** 1 Department of Hematology, Institute of Hematology and Transfusion Medicine, 02-776 Warsaw, Poland; asalomon@ihit.waw.pl (A.S.-P.); malenda.agata@gmail.com (A.M.); 2 Department of Hematology and Transfusion Medicine, Center of Postgraduate Medical Education, 02-776 Warsaw, Poland; 3 Department of Diagnostic Hematology, Institute of Hematology and Transfusion Medicine, 02-776 Warsaw, Poland; szumann@gmail.com (A.S.-C.); monika.prochorec@interia.pl (M.P.-S.); 4 Department of Gastroenterology, Hepatology and Clinical Oncology, Centre of Postgraduate Medical Education, 02-781 Warsaw, Poland; p.wieszczy@gmail.com; 5 Department of Experimental Hematology, Institute of Hematology and Transfusion Medicine, 02-776 Warsaw, Poland; filip.garbicz@gmail.com (F.G.); imisiewiczk@ihit.waw.pl (I.M.-K.); pjuszczynski@ihit.waw.pl (P.J.)

**Keywords:** DNA damage-binding protein 1, cereblon, cullin 4a, thalidomide, lenalidomide, immunomodulatory drugs, multiple myeloma

## Abstract

Immunomodulatory drugs (IMiDs) are effective in the treatment of multiple myeloma (MM), myelodysplastic syndrome with deletion of chromosome 5q and other haematological malignancies. Recent studies showed that IMiDs bind to cereblon (CRBN), a substrate receptor of the CRL4–CRBN complex, to induce the ubiquitination and degradation of IKZF1 and IKZF3 in MM cells, contributing to their anti-myeloma activity. We aimed to determine whether the CRL4–CRBN complex proteins’ expression predicts the prognosis of MM patients treated with IMiDs. Here, we evaluated the expression of CRL4–CRBN complex proteins and their downstream targets with immunohistochemistry (IHC) staining in 130 bone marrow samples from MM patients treated with thalidomide or lenalidomide-based regimens. We found that the expression of CRBN and CUL4A was associated with the superior IMiD-based treatment response (*p* = 0.007 and *p* = 0.007, respectively). Moreover, the CUL4A expression was associated with improved PFS (HR = 0.66, 95% CI 0.44–0.99; *p* = 0.046) and DDB1 expression showed a negative impact on OS both in the univariate (HR = 2.75, 95% CI 1.65–4.61; *p* = 0.001) and the multivariate (HR 3.67; 95% CI 1.79–7.49; *p* < 0.001) analysis. Overall, our data suggest that the expression of DDB1, CUL4A and CRBN assessed by IHC predicts the clinical course of MM patients and identifies patients with a high probability of responding to IMiD-based therapy.

## 1. Introduction

Multiple myeloma (MM) is the third most common haematologic malignancy in the European Union, with approximately 33,000 new cases and 20,000 deaths annually [1]. Despite the impressive therapeutic progress that has occurred in recent decades, the development of drug resistance is typical during the clinical course of MM, and most patients eventually relapse and require further therapy [2]. The introduction of thalidomide, a first-in-class immunomodulatory drug (IMiD), in 2006 was one of the milestones in MM therapy history. Thalidomide and its newer derivatives such as lenalidomide and pomalidomide, along with proteasome inhibitors, are the backbone of most combination regimens used in the treatment of MM. In Poland and other European countries, thalidomide-based regimens are the most common therapy for young and fit newly diagnosed MM patients; lenalidomide and next-generation IMiDs are available for relapsed/refractory groups of patients. IMiDs have been shown to have a pleiotropic anti-cancer effect, including anti-angiogenic, anti-proliferative, anti-inflammatory and immune-modulatory effects [3]. The primary molecular target for IMiDs is cereblon (CRBN), which functions as a substrate receptor in the cullin-4 RING E3 ubiquitin ligase (CRL4–CRBN) complex co-formed by other proteins, such as DNA damage-binding protein 1 (DDB1), cullin 4A (CUL4A), and regulator of cullins-1 (ROC1) [4,5]. By binding CRBN, IMiDs modify the substrate specificity of the CLR4–CRBN complex leading to ubiquitination and degradation of the lymphoid transcription factors Ikaros (IKZF1) and Aiolos (IKZF3), and the casein kinase 1α (CK1α) [6,7,8]. Therefore, the degradation of IKZF1 and IKZF3 decreases the expression of IRF4 and its downstream target MYC, resulting in growth inhibition of multiple myeloma cells [7,9,10]. In T cells, IKZF1 and IKZF3 are transcriptional repressors of the IL-2 gene [11,12]; their degradation therefore releases repression and causes an increased production of IL-2, leading to T and NK cell activation [13].

Recent studies have established a correlation between CRBN expression levels and clinical response to IMiD treatment, however the results are non-conclusive. High expression of CRBN in patients receiving thalidomide maintenance for 2 years was associated with longer PFS in the HOVON-65/GMMG-HD4 trial, while no association was noted in those on bortezomib maintenance [14]. CRBN high expression has also been shown to enhance lenalidomide therapy’s effects in terms of treatment response [15]. Conversely, the loss of CRBN protein and CRBN mRNA level led to lenalidomide resistance in myeloma cells and a poor outcome in MM patients [9,16,17].

In this study, we separately evaluated the expression of CRL4–CRBN complex proteins (CRBN, DDB1, CUL4A) and their downstream targets (IKZF1, IKZF3) with IHC staining, using FFPE bone marrow samples from 130 MM patients treated with IMiDs. In the samples from MM patients, we aimed to compare the expression of CRBN-CRL4 complex proteins and their downstream targets in the malignant plasma cells and assess their potential correlation with the MM patients’ clinical course, despite the type of IMiD therapy or stage of the disease.

## 2. Materials and Methods

### 2.1. Patients and Bone Marrow Samples

The study retrospectively analysed 130 patients diagnosed with MM from 2010 to 2015 at the Institute of Haematology and Transfusion Medicine, Warsaw, Poland. The following data were obtained about the patients: age, sex, disease stage according to the International Staging System (ISS), type of monoclonal protein and its concentration, free light chain (FLC) type and FLC ratio, haemoglobin, calcium and creatinine concentration, the presence of osteolytic lesions, the percentage of plasma cell infiltration in the bone marrow. All patients received therapy with IMiDs: 81 with thalidomide for newly diagnosed MM (NDMM) and 49 with lenalidomide for relapsed/refractory disease (RRMM). Duplicate records of the same patients were rejected from the analysis. IHC evaluation was performed just before the initiation of treatment with thalidomide or lenalidomide-based regimens. The study was conducted according to the Declaration of Helsinki, and the protocol was approved by the Ethics Committee of the Institute of Haematology and Transfusion Medicine, Warsaw, Poland.

### 2.2. Treatment Response

The treatment responses, progression-free survival (PFS) and overall survival (OS), were evaluated according to the International Myeloma Working Group panel consensus (19, 20). The study follow-up was defined as the time from the MM diagnosis to death, of any cause, or to the date of last observation with the cut-off date of 6 August 2018.

### 2.3. Immunohistochemistry Staining

All trephine samples had an established diagnosis according to the histopathological recommendations (WHO and International Myeloma Working Group) for monoclonal plasma cell proliferative disorders. The trephine biopsies were treated with a combined fixative and decalcifier solution (40% formaldehyde, glacial acetic acid, NaCl, H_2_O distilled) and then routinely processed staining with haematoxylin and eosin. Immunohistochemistry was performed using an automated immunohistochemical stainer (Dako Denmark A/S, Glostrup, Denmark), and mono- and polyclonal antibodies were applied, including anti-DDB1 (clone: LS-B3138, 1:500 LSBio, Lifespan Biosciences, Seattle, WA, USA), Aiolos/IZKF3 (clone: NBP2-24495, 1:50, NovusBio, Novus Biologicals, Abingdon, UK), CUL4a (clone: NBP1-44439, 1:300, NovusBio, Novus Biologicals, Abingdon, UK), anti-CRBN (cloneCRBN65, 1:2000, Celgene Corporation, New York, NY, USA) and anti-Ikaros/IZK1 (clone: ab26083, 1:50, Abcam, Cambridge, UK). Plasma cells were visualised by the reaction with the CD138 antibody (clone: MI15, RTU, Dako Omnis, Agilent, Santa Clara, CA, USA). All stainings were performed according to the manufacturer’s instructions, and the EnVision Detection System (Dako, Denmark A/S, Glostrup, Denmark) was used for signal detection. A positive staining controls were applied for each antibody: anti-Ikaros/IZKF1–tonsil, anti-DDB1–adrenal gland, anti-Aiolos/IKZF3–tonsil, CUL4a–colon, anti-CRBN–liver. Negative (isotype) control stainings were performed using a ready to use FLEX Negative Mouse Control (a cocktail of mouse IgG1, IgG2a, IgG2b, IgG3 and IgM; code nr IR750; Dako Denmark A/S, Glostrup, Denmark). All neoplastic cells were scored independently by two experienced haematopathologists (M.P-S. and A.S-C.) for each target protein’s immunoreactivity based on staining intensity and the percentage of cells staining positively. Based on in-house validation, the cut-offs (>30% for anti-DDB1, Aiolos/IKZF3, anti-CRBN, ≥30% for DDB1 and ≥80% for anti-Ikaros/IKZF1) of strongly and/or intermediate positive neoplastic plasma cells were implemented as a final distinction between the positive and negative results of the staining. Two independent pathologists reviewed the samples, and discrepancies were revised to determine the consensus result. All microphotographs were taken by a microscope DP72 Olympus BX63 camera (Olympus, Tokyo, Japan).

### 2.4. Statistical Analysis

Categorical variables were compared using the chi-squared test or the Fisher test, depending on the number of observations in each 2-by-2 table. Continuous variables were compared using the t-Student test if they followed normal distribution, or the Wilcoxon test if they did not follow normal distribution. The distribution of the variables was checked by plotting histograms. A survival function with 95% confidence intervals was estimated using the Kaplan–Meier method. To estimate the hazard ratios and 95% confidence intervals, the Cox proportional hazard model was used. For the multivariable models, the forward stepwise variable selection was applied at a 0.15 significance level. All tests were two-sided and were performed at a 0.05 significance level. All analyses were performed using Statistica software, ver. 13.1.

## 3. Results

### 3.1. Patients’ Characteristics

The patients’ clinical characteristics are summarised in Table 1. Among the 130 patients included in the analysis, 81 (62%) were treated with thalidomide in the frontline setting and 49 (38%) received lenalidomide for RRMM. The median age of the patients at the initiation of the IMiD-based treatment was 62.5 years (range, 32–85 years), with female predominance (52 men and 78 women). The isotype of monoclonal proteins was as follows: IgG in 86 patients (66%), IgA in 29 patients (22%), light chains in 14 patients (11%) and IgE protein in 1 patient (1%). The distribution according to the ISS was 18%, 32% and 28%, with a score of 1, 2 and 3, respectively (missing data for 22% of patients). A decreased haemoglobin level (<10 g/dL) was observed in 69 patients (53%), hypercalcemia (>2.55 mmol/L) was noticed in 21 patients (16%) and renal impairment was noted in 8 patients (6%). Frontline treatment with thalidomide was combined with cyclophosphamide and dexamethasone in the majority of patients (*n* = 62, 77% of thalidomide-based regimens); lenalidomide was applied in combination with dexamethasone for all RRMM cases. A comparison of the patients’ baseline characteristics revealed no significant differences between the IMiD-based treatment groups, except for higher rates of female patients (66% vs. 49%) and higher rates of osteolytic lesions in the thalidomide group (64% vs. 28%), Supplement Table S1. The final analyses were performed with data from all of the patients treated with IMiD-based regimens.

### 3.2. CRL4–CRBN Complex Proteins IHC Staining

The IHC staining for all evaluated proteins is shown in Supplement Figures S1 and S2. According to the predefined cut-off values, the positivity (+) of CRBN, CUL4A, DDB1, IKZF1 and IKZF3 was observed in 54%, 51%, 49%, 71% and 54% of cases, respectively. We evaluated the internal associations between the proteins’ expression involved in the CRL4–CRBN complex, and positive ones were observed for pairs: CUL4A–IKZF3 (*p* = 0.023) and DDB1–IKZF1 (*p* = 0.007). There were no other significant associations for the CRBN–CRL4 complex or its activity-dependent transcriptional factors (IKZ1, IKZF3).

### 3.3. CRL4–CRBN Complex Proteins’ Associations with Clinical Features

The group of patients with CRBN^(+)^ had a significantly higher serum concentration of monoclonal protein (median 4.1 g/dL vs. 2.7 g/dL, *p* = 0.006), β2-microglobulin (median 4.6 mg/L vs. 3.9 mg/L, *p* = 0.047) and a haemoglobin concentration lower than 10 g/dL (*p* = 0.030) before treatment initiation. The DDB1^(+)^ was associated with elevated β2-microglobulin (median 5.04 g/dL vs. 3.94 g/dL, *p* = 0.016), a haemoglobin concentration below 10 g/dL (*p* = 0.036) and a shorter IMiD exposure (median number of cycles 5.7 vs. 6.4; *p* = 0.022). A high expression of proteins involved in B-cell maturation and a switch of immunoglobulin classes—IKZF1 and IKZF3—were associated with the isotype of serum monoclonal protein (*p* = 0.040 and *p* = 0.0001, respectively).

### 3.4. CRBN and CUL4a as Predictive Markers of IMiD-Based Treatment Response

**Figure 1:**
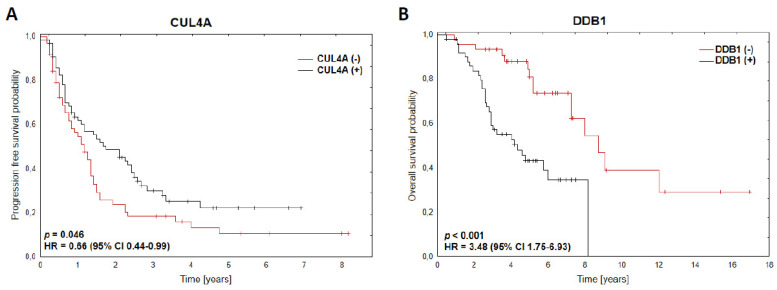
(**A**) Kaplan–Meier survival curves for PFS according to CUL4A expression (classified as positive and negative results of IHC staining), *n* = 130. (**B**) Kaplan–Meier survival curves for OS according to DDB1 expression (classified as positive and negative results of IHC staining), *n* = 97.

Among those analysed, all patients’ data had a median number of cycles, with IMiD-based treatment, of 6 (range, 1–56). The patients with CRBN^(+)^ had a significantly superior response to treatment than those with CRBN^(−)^ (ORR ≥ PR vs. SD/PD, *p* = 0.012; ORR ≥ VGPR vs. PR/SD/PD, *p* = 0.032, Table 2). The CUL4A^(+)^ was also associated with a better response to treatment than the CUL4A^(−)^ group of patients (ORR ≥ PR vs. SD/PD, *p* = 0.007; ORR ≥ VGPR vs. <VGPR, *p* = 0.027, Table 2). There were no significant differences observed in the expression of DDB1, IKZF1 and IKZF3 in terms of treatment response. After a median follow-up of 4.75 years (range, 0.5–16.9), only CUL4A^(+)^ impacts progression-free survival (HR = 0.66, 95% CI 0.44–0.99; *p* = 0.046), Figure 1A.

### 3.5. DDB1 as a Prognostic Marker of MM Patients’ Survival upon IMiD-Based Treatment

**Figure 2:**
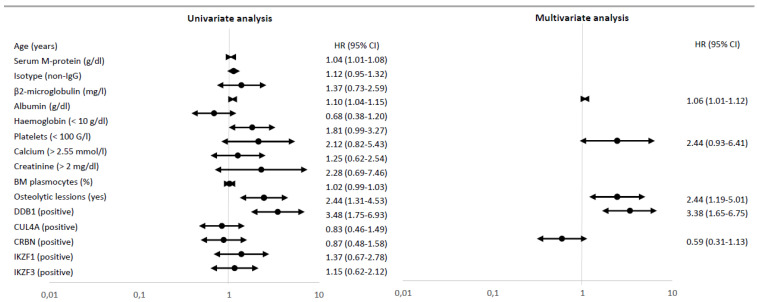
Univariate and multivariate Cox regression analyses for OS (*n* = 97). If the hazard ratio (HR) is greater than 1, then the predictor is associated with an increased risk of death.

The median overall survival rate of the analysed group was 4.25 years (range, 0.5–16.9). The univariate analyses revealed that a poor overall survival rate was associated with DDB1^(+)^ (HR = 3.48, 95% CI 1.75–6.93; *p* < 0.001, Figure 1B), the presence of osteolytic lesions (HR 2.44; 95% CI 1.31–4.53, *p* = 0.005), an older age (HR 1.04, 95% CI 1.01–1.08, *p* = 0.008) and a higher β2-microglobulin concentration (HR 1.10, 95% CI 1.04–1.15, *p* < 0.001) at the initiation of IMiD-based treatment (Figure 2). Multivariate Cox analysis using the forward stepwise elimination method and including all variables assessed in univariate analysis (age, serum M-protein, the isotype of immunoglobulin, bone marrow plasma cells, β2-microglobulin, albumin, creatinine, calcium, haemoglobin, platelets and expression of CRL4–CRBN complex proteins) confirmed that a poor prognosis was independently predicted by DDB1^(+)^ (HR 3.38; 95% CI 1.65–6.75; *p* < 0.001), presence of osteolytic lesions (HR 2.44; 95% CI 1.19–5.01, *p* = 0.015) and β2-microglobulin concentration (HR 1.06; 95% CI 1.01–1.12, *p* = 0.026), Figure 2. As a result, DDB1 expression was confirmed as the significant prognostic marker in MM patients.

## 4. Discussion

To the best of our knowledge, this is the first study showing that the expression of DDB1 and CUL4A assessed by routine, diagnostic IHC evaluation of bone marrow samples is associated with the outcome of multiple myeloma patients treated with IMiDs. Our analyses also revealed that CRBN expression impacts the superior response to thalidomide or lenalidomide-based treatment in line with previously published data.

It was shown that positive CRBN IHC staining is associated with a superior response rate in patients with newly diagnosed and RRMM treated with thalidomide–dexamethasone (TD) and lenalidomide–dexamethasone (LD) [18]. However, Dimopoulos et al. used CRBN IHC staining in FFPE bone marrow samples of 23 MM patients and found no correlation between the CRBN protein level and the sensitivity or intrinsic resistance to lenalidomide-based therapy [19]. These conflicting results should be considered in the context of the limited ability of commercially available assays to measure the CRBN protein level in MM reliably. Gandhi et al. have developed a novel CRBN monoclonal antibody CRBN65 and have shown its superiority over other commercially available antibodies in IHC staining [16]. Using the CRBN65 antibody, we found that MM patients with CRBN^(+)^ have a superior response rate to thalidomide- or lenalidomide-based therapy compared to those with CRBN^(-)^ (ORR ≥PR vs. SD/PD, *p* = 0.012; ORR ≥ VGPR vs. PR/PD/SD, *p* = 0.032). In line with our findings, several studies using different approaches to assessing CRBN gene expression, such as real-time PCR [15,20] or gene expression profiling [21], have demonstrated the predictive value of CRBN gene expression in MM patients treated with TD, LD or pomalidomide with dexamethasone. In general, across all of these studies, higher CRBN gene expression was associated with a superior response rate to treatment with IMiD monotherapy [21], or in combination with dexamethasone [15,20,21]. Conversely, low CRBN gene expression determined unresponsiveness to the abovementioned therapy [21]. This recent study also showed that MM patients with hyperdiploid karyotype have a better response rate with IMiD-based therapy and achieve a longer time to next treatment when IMiD-based therapy is applied, compared to their non-hyperdiploid counterparts [22]. It is postulated that it may be associated with the higher expression of CRBN which characterises hyperdiploid-myeloma patients [21].

Here, we show that the CUL4A protein level, a component of the CRL4–CRBN complex, has predictive value in MM patients treated with thalidomide- or lenalidomide-based therapy with higher response rates (ORR ≥ PR vs. SD/PD, *p* = 0.007; ORR ≥ VGPR vs. <VGPR, *p* = 0.027), translated into a favourable PFS (HR = 0.66, 95% CI 0.44–0.99; *p* = 0.046). CUL4A has been shown to play an oncogenic role in various cancer types [23,24,25]. In MM, CUL4A promotes proliferation, invasion and migration of plasma cells [26]. The positive correlation between the high expression of CUL4A and thalidomide sensitivity was also demonstrated in prostate cancer cell lines [27].

We also show here that DDB1 expression is independently associated with poor OS in both univariate and multivariate analyses (HR 3.38; 95% CI 1.65–6.75; *p* < 0.001). To the best of our knowledge, this is the first report showing the prognostic impact of the DDB1 protein level on the survival of MM patients. DDB1 is essential for DNA repair and plays an important role in many signaling pathways related to carcinogenesis. Moreover, DDB1 overexpression in malignant cells may lead to resistance to anti-cancer therapy [28]. Recently, a high expression of DDB1 was identified as a poor prognostic factor in pancreatic cancer [29]. In our study, CRBN^(+)^ and DDB1^(+)^ were associated with clinical features corresponding to the high burden of MM disease (a higher serum concentration of monoclonal protein or β2-microglobulin, as well as a haemoglobin concentration lower than 10 g/dL). However, there were no significant associations between the expression of those two proteins. The superior response rate in the CRBN^(+)^ group of patients confirms the established mechanism of IMiD action via direct interaction of IMiDs and CRBN. The DDB1 negative impact on the OS and the lack of associations with response rates or PFS may indicate that this component of CRL4–CRBN is more involved in other signaling pathways related to MM cell survival. Therefore, further studies are needed to gain more insight into the DDB1 mechanism of action in MM cells.

The relationship between the expression of CRL4–CRBN downstream targets and IMiD activity remains unclear and inconclusive. Lu et al. [8] found that some MM cell lines with a higher expression of IKZF1 or IKZF3 showed resistance to the lenalidomide. In contrast, Zhu et al. [30] showed that low IKZF1 transcript levels were correlated with a poor response to IMiDs. They also found that higher IKZF1, but not IKZF3, gene expression was associated with better OS. In contrast, Pourabdollah et al. [31] showed that especially IKZF3 expression is correlated with a better outcome in refractory MM patients treated with lenalidomide. In our study, we did not observe any associations of IKZF1/IKZF3 expression in the response rate or MM patients’ survival. Therefore, the significant associations of IKZF1^(+)^ and IZKF3^(+)^ with the isotype of serum monoclonal protein (*p* = 0.040 and *p* = 0.0001, respectively) confirm their contribution to switch immunoglobulin classes during B-cell maturation [32,33] and verify the reliability of applied assays.

There is an increasing amount of evidence indicating that IMiD-resistance arises, at least in part, from the acquisition and selection of mutations in genes coding protein downstream or components of the CLR4–CRBN complex, especially *CRBN* and *IKZF1* [34,35]. Consistently, a large-scale genomic and transcriptomic analysis including patients with treatment-naïve-, lenalidomide-refractory and pomalidomide-refractory MM recently showed an increase in the frequency of CRBN aberrations (namely, point mutations, copy number variants, structural variations and exon 10 spliced transcript) with progressive IMiD exposure. Eventually, alterations in CRBN were found in one third of the pomalidomide-resistant patients [36]. It needs to be emphasised here that lenalidomide-refractory patients who harbored CRBN aberrations had a significantly shorter PFS when pomalidomide-based therapy was applied.

However, one should be aware of several limitations of this study. First, there is controversy regarding the most appropriate IHC antibodies, testing method and scoring system for evaluating protein expression using IHC in bone marrow samples. Thus, it would be important to establish replicable methods for the quantitative evaluation ofCRL4–CRBN complex protein expression in FFPE bone marrow samples from MM patients. Second, to provide a sufficient sample size, this study was performed in a retrospective manner, and clinical data were collected from different treatment approaches (both thalidomide- and lenalidomide-based regimens) applied for newly diagnosed or relapsed/refractory MM patients. Third, cytogenetic and molecular data were not available for an analysed group of MM patients. That additional information might produce more powered results in terms of response to IMiD treatment or survival estimations.

In the era of multiple treatment options for patients with MM, having reliable predictive tools is crucial in clinical practice. Our results add some novelty to the understanding of the prognostic and predictive role of some protein components of the CLR4–CRBN complex in MM patients treated with IMiDs. Nevertheless, given the complexity of the molecular mechanisms involved in sensitivity or resistance to IMiD-based therapy, the predictive and prognostic role of the CLR4–CRBN complex cannot be reliably established without a comprehensive analysis and understanding of genetic, transcriptomic and proteomic data obtained from treatment-naïve MM patients, as well as those who have become resistant to thalidomide, lenalidomide and pomalidomide.

In conclusion, this study revealed that the expression of CUL4A and CRBN assessed by routine IHC in FFPE bone marrow samples needs further evaluation as a potential predictive factor for MM patients treated with IMiDs. Moreover, DDB1 expression was found as an independent prognostic factor for the overall survival of patients with MM. Therefore, assessment of the CRL4–CRBN expression in bone marrow samples may improve identifying the MM patients who most benefit from IMiD-based therapies, despite the type of immunomodulatory drug or stage of the disease.

## Figures and Tables

**Table 1:** Overall clinical patients’ characteristics. Data are shown as a number (percentage) or median (interquartile range). Abbreviations: ISS—International Staging System; FLC—free light chain; BM—bone marrow; IMiD—immunomodulatory drug.

Parameter		Overall (*n* = 130)
Age, years		62.5 (32–85)
Sex	male	52 (40%)
	female	78 (60%)
Isotype of M-protein	IgG	86 (66%)
	IgA	29 (22%)
	IgE	1 (1%)
	FLC	14 (11%)
ISS	stage I	23 (18%)
	stage II	42 (32%)
	stage III	36 (28%)
	no data	29 (22%)
Albumin, g/dL		3.59 (±0.54) *n* = 128
β2-microglobulin, mg/L		4.43 (1.56–26.15) *n* = 100
Serum M-protein, g/dL		3.6 (±2.01) *n* = 125
Serum FLC ratio	<100	43 (33%)
	≥100	43 (33%)
	no data	44 (33%)
Haemoglobin, g/dL	˂10	69 (53%)
	≥10	61 (47%)
Calcium, mmol/L	≤2.55	108 (83%)
	˃2.55	21 (16%)
	no data	1 (1%)
Creatinine, mg/dL	≤2 mg/dL	122 (94%)
	˃2 mg/dL	8 (6%)
Osteolytic lesions	yes	66 (50%)
	no	58 (45%)
	no data	6 (5%)
BM plasma cells, %		67.5 (12.5–95)
IMiD	thalidomide	81 (62%)
	lenalidomide	49 (38%)
Cycles of IMiD-based treatment		6 (1–56)

**Table 2:** Quality of IMiD-based treatment response stratified by CRBN and CUL4A expression. Abbreviations: CR—complete response; VGPR—very good partial response; PR—partial response; SD—stable disease; PD—progressive disease; ORR—overall response rate; *p*-Value < 0.5 is bolded.

	CRBN^(−)^ *n* = 60	CRBN^(+)^ *n* = 70	*p*-Value	CUL4A^(−)^ *n* = 64	CUL4A^(+)^ *n* = 66	*p*-Value
	*n* (%)	*n* (%)	*p* = 0.105	*n* (%)	*n* (%)	***p*** = **0.011**
CR	4 (7)	7 (10)		1 (1)	10 (15)	
VGPR	8 (13)	19 (27)		12 (19)	15 (23)	
PR	19 (32)	25 (36)		20 (31)	24 (36)	
SD	20 (33)	12 (17)		19 (30)	13 (20)	
PD	9 (15)	7 (10)		12 (19)	4 (6)	
ORR (≥PR)	31 (52)	51 (73)	***p*** = **0.0126**	33 (52)	49 (74)	***p*** = **0.007**
ORR (<PR)	29 (48)	19 (27)		31 (48)	17 (26)	
ORR (≥VGPR)	12 (20)	26 (37)	***p*** = **0.0321**	13 (20)	25 (38)	***p*** = **0.027**
ORR (<VGPR)	48 (80)	44 (63)		51 (80)	41 (62)	

## Data Availability

The data presented in this study are available on request from the corresponding author.

## Supplementary Materials

The following are available online at https://www.mdpi.com/article/10.3390/jcm10122683/s1. Table S1. Overall clinical patients’ characteristics with detailed division into thalidomide and lenalidomide-based treatment; Figure S1. The immunohistochemical characterization of malignant plasma cells; Figure S2. Positive and negative DDB1 IHC profiles in multiple myeloma
