# Supplementary material for: The CRBN, CUL4A and DDB1 Expression Predicts the Response to Immunomodulatory Drugs and Survival of Multiple Myeloma Patients
Source: J Clin Med. 2021 Jun 18;10(12):2683. doi: 10.3390/jcm10122683 (PMC8235391; doi:10.3390/jcm10122683)

Supplementary materials

Supplementary Table S1. Overall clinical patients' characteristics with detailed division into thalidomide and lenalidomide-based treatment.
Abbreviations: ISS - International Staging System; FLC – free light chain; BM – bone marrow; IMiD – immunomodulatory drug; LEN – lenalidomide; THAL – thalidomide; LD – lenalidomide, dexamethasone; TD – thalidomide, dexamethasone; CTD – cyclophosphamide, thalidomide, dexamethasone; VTD – bortezomib, thalidomide, dexamethasone; MPT – melphalan, prednisone, thalidomide

**Parameter**		**LEN-based treatment (n=49)**	**THAL-based treatment (n=81)**	**P-value**
**Age, years**		61.4 (32-80)	63 (47-85)	0.226
**Sex**	male	25 (51%)	27 (34%)	**0.046**
	female	24 (49%)	54 (66%)	
**Isotype of M-protein**	IgG	33 (68%)	53 (65%)	0.279
	IgA	8 (16%)	21 (26%)	
	IgE	1 (2%)	0	
	FLC	7 (14%)	7 (9%)	
**ISS**	stage I	12 (24%)	11 (12%)	0.052
	stage II	12 (24%)	30 (37%)	
	stage III	8 (16%)	28 (36%)	
	no data	17 (36%)	12 (15%)	
**Albumin, g/dl**		3.67 (±0.54)	3.56 (±0.54) n=79	0.363
**β2-microglobulin, mg/l**		3.7 (1.56, 18.64) n=31	4.6 (1.75, 26.15) n=69	0.073
**Serum M-protein, g/dl**		3.53 (±1,80) n=45	3.63 (±2,12)	0.723
**Serum FLC ratio**	<100	14 (29%)	29 (36%)	0.267
	>=100	19 (39%)	24 (30%)	
	no data	16 (32%)	28 (34%)	
**Haemoglobin, g/dl**	< 10	18 (37%)	43 (53%)	0.071
	≥ 10	31 (63%)	38 (47%)	
**Calcium, mmol/l**	≤ 2,55	42 (86%)	66 (82%)	0.631
	> 2,55	7 (14%)	14 (17%)	
	no data	0	1 (1%)	
**Creatinine, mg/dl**	≤ 2 mg/dl	47 (96%)	75 (93%)	0.444
	> 2 mg/dl	2 (4%)	6 (7%)	
**Osteolytic lessions**	yes	14 (28%)	52 (64%)	**0.001**
	no	29 (59%)	29 (36%)	
	no data	6 (13%)	0	
**BM plasma cells, %**		65 (17.5-95) n=48	70 (12.5-95) n=80	
**Cycles of IMiD-based treatment (median, range)**	6 (1-56)	9.5 (3-56)	6 (1-24)	

**Prior lines of treatment**	n/a	4 (1-8)	n/a	
**Treatment regimen**	LD	49 (100%)		
	TD		2 (2%)	
	CTD		62 (77%)	
	VTD		2 (2%)	
	MPT		15 (19%)	

Supplementary Figure S1. The immunohistochemical characterization of malignant plasma cells (#5 case): A – HE; B – CD138; C – positive IZKF3; D – positive CUL4A; E – positive IKZF1; F – positive CRBN; G – negative DDB1; (all photographs in 600x magnification).

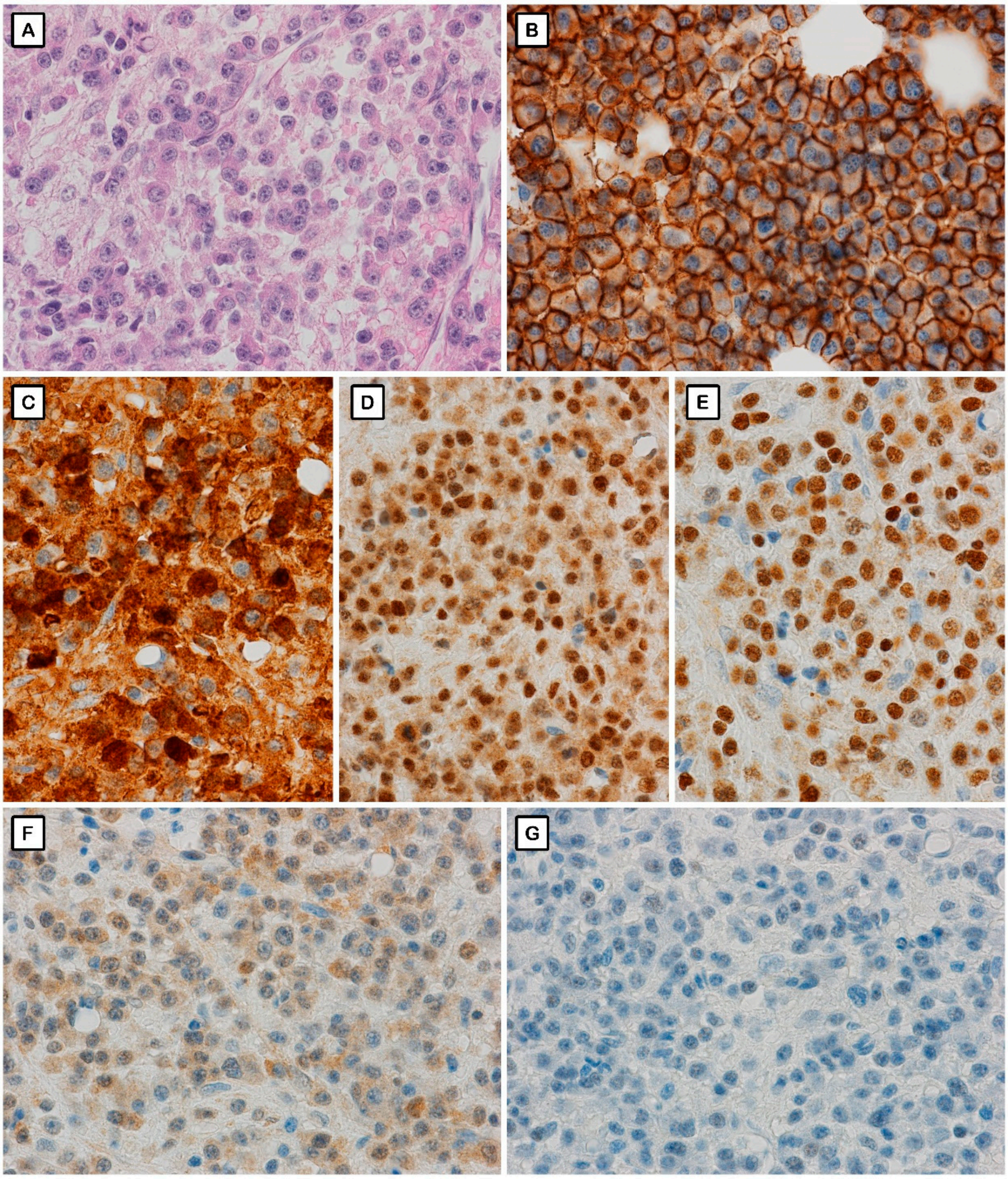

Supplementary Figure S2. Positive and negative DDB1 IHC profiles in multiple myeloma; case #1: A – HE; B – CD138; C – DDB1 positive (>30% of strongly positive cells); case #2: D - HE; E – CD138; F – DDB1 negative (≤30% of strongly positive cells); (arrow – atypical plasma cell with triple nuclei; all photographs in 600x magnification).

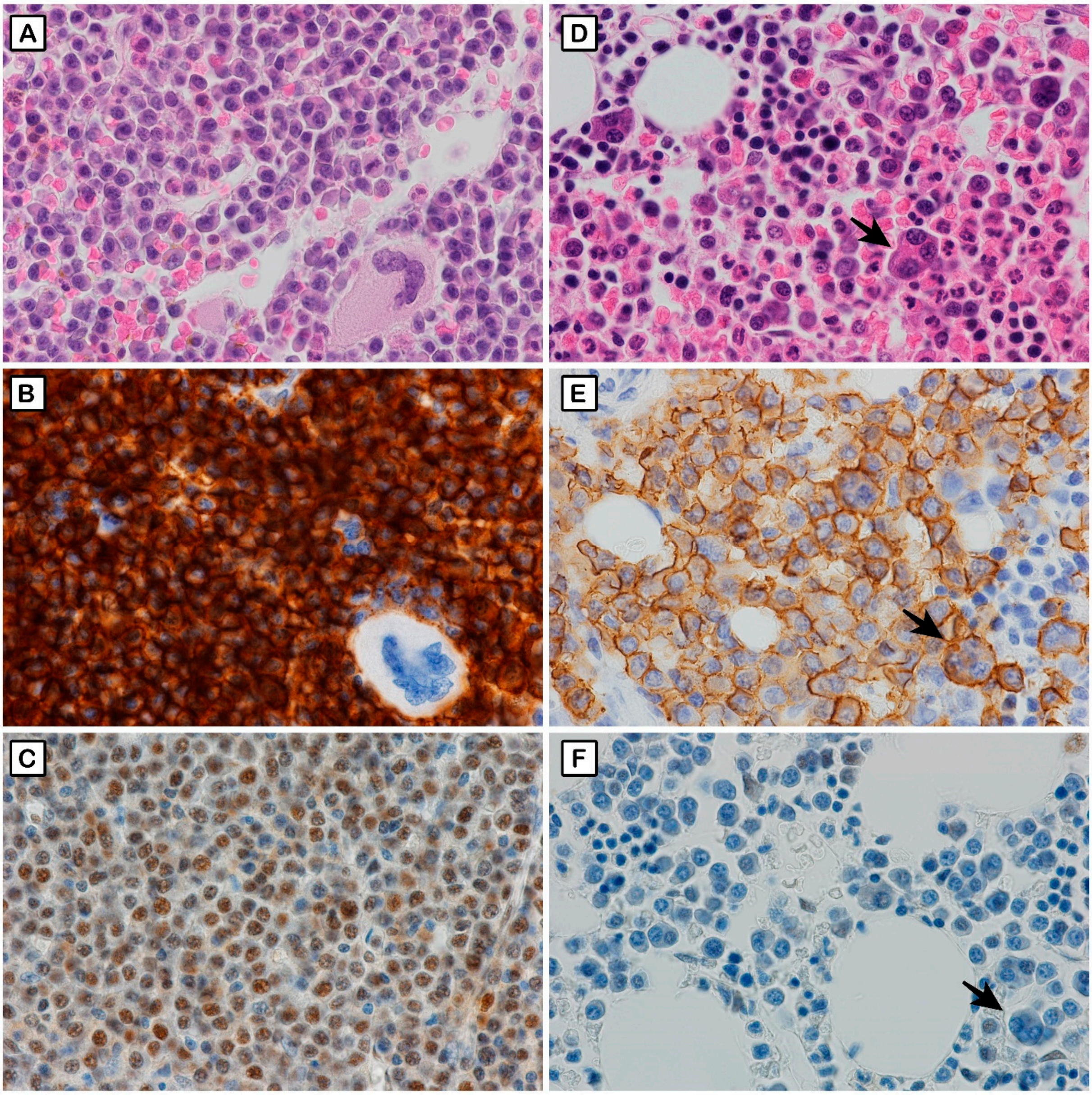

Supplement: Supplementary file 1 [file jcm-10-02683-s001.zip › jcm-1261105-SI.pdf]
